# Correction: Nam et al. Identification of Thiazolo[5,4-*b*]pyridine Derivatives as c-KIT Inhibitors for Overcoming Imatinib Resistance. *Cancers* 2023, *15*, 143

**DOI:** 10.3390/cancers16112151

**Published:** 2024-06-06

**Authors:** Yunju Nam, Chan Kim, Junghee Han, SeongShick Ryu, Hanna Cho, Chiman Song, Nam Doo Kim, Namkyoung Kim, Taebo Sim

**Affiliations:** 1 KU-KIST Graduate School of Converging Science and Technology, Korea University, 145 Anam-ro, Seongbuk-gu, Seoul 02841, Republic of Korea; 2 Severance Biomedical Science Institute, Graduate School of Medical Science, Yonsei University College of Medicine, 50 Yonsei-ro, Seodaemun-gu, Seoul 03722, Republic of Korea; 3 Chemical Kinomics Research Center, Korea Institute of Science and Technology, 5 Hwarangro 14-gil, Seongbuk-gu, Seoul 02792, Republic of Korea; 4 Voronoibio Inc., 32 Songdogwahak-ro, Yeonsu-gu, Incheon 21984, Republic of Korea

The authors would like to make a correction to the previous article [1]. In Figure 6A, an error was introduced in the preparation of this figure for publication.

The error included:

The image treated with **6r** (0.05 μM) in 0 h was mistakenly replaced by **6r** (0.005 μM). The authors have provided the corrected version of the Figure 6 below. The authors apologize for any inconvenience caused and state that the scientific conclusions are unaffected. This correction was approved by the Academic Editor. The original publication has also been updated.

**Figure 6:**
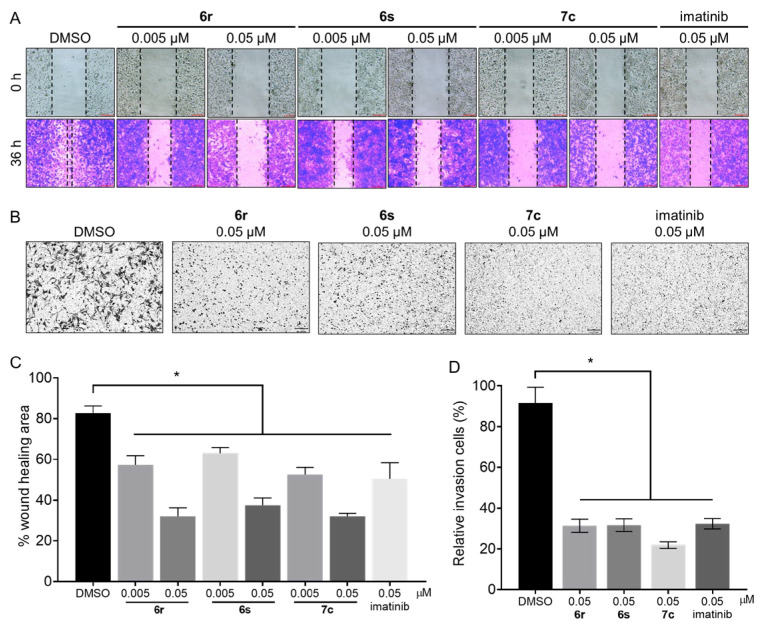
Effects of **6r**, **6s**, **7c**, and imatinib on migration and invasion. (**A**) Cell migration of GIST-T1 cells was inhibited by treatment (36 h) with **6r**, **6s**, and **7c** (0.005 μM and 0.05 μM). (**B**) Invasion of GIST-T1 cells was inhibited by treatment (48 h) with **6r**, **6s**, **7c**, and imatinib (0.05 μM). (**C**,**D**) Percentage wound healing area and relative invasion cells were calculated using ImageJ. (average ± S.D., n = 3 one-way ANOVA and Tukey’s multiple comparisons test; * *p* < 0.05).
