# Supplementary material for: Identification of Thiazolo[5,4-b]pyridine Derivatives as c-KIT Inhibitors for Overcoming Imatinib Resistance
Source: Cancers (Basel). 2022 Dec 26;15(1):143. doi: 10.3390/cancers15010143 (PMC9817970; doi:10.3390/cancers15010143)
Supplement: Supplementary file 1 [file cancers-15-00143-s001.zip › File S1_c-KIT_tbsim_12-26-2022.pdf]

Supplementary Materials

Identification of Thiazolo[5,4-*b*]pyridine Derivatives as c-KIT Inhibitors for Overcoming Imatinib Resistance

Yunju Nam[1,2,3,†], Chan Kim[2,†], Junghee Han[1,2,3], SeongShick Ryu[1,2,3], Hanna Cho[2], Chiman Song[3], Nam Doo Kim[4], Namkyoung Kim[1,2,3], Taebo Sim[1,2,3,*]

[1]KU-KIST Graduate School of Converging Science and Technology, Korea University, 145 Anam-ro, Seongbuk-gu, Seoul, 02841, Republic of Korea

[2]Severance Biomedical Science Institute, Graduate School of Medical Science, Brain Korea 21 Project, Yonsei University College of Medicine, 50 Yonsei-ro, Seodaemun-gu, Seoul, 03722, Republic of Korea

[3]Chemical Kinomics Research Center, Korea Institute of Science and Technology, 5 Hwarangro 14-gil, Seongbuk-gu, Seoul, 02792, Republic of Korea

[4]Voronoibio Inc., 32 Songdogwahak-ro, Yeonsu-gu, Incheon, 21984, Republic of Korea

[†]These authors contributed equally to this work.

[*]Author to whom correspondence should be addressed.

Table of contents

Synthesis and chemical characterization……………………………………………………………………….S2

Synthesis and chemical characterization

6-Bromothiazolo[5,4-*b*]pyridine-2-amine (2). To a solution of 3-amino-5-bromo-2-chloropyridine (1.5 g, 7.284 mmol) in concentrated HCl (15 ml) was added potassium thiocyanate (1.413 g, 14.57 mmol). The mixture was heated at 100 °C for 12 h. After completion, the mixture was added ice-cold water and neutralized with saturated sodium carbonate solution. The resulting solid was filtered, dried to afford **2** (1.26 g, 75 %) as a green solid, and used for the next step without purification. ^{1}H NMR (400 MHz, DMSO-d_6) δ 8.18 (d, J = 2 Hz, 1H), 8.04 (s, 2H), 7.85 (d, J = 2 Hz, 1H); ^{13}C NMR (100 MHz, DMSO-d_6) δ 167.12, 154.10, 148.30, 141.42, 125.39, 117.10; LRMS (ESI) *m/z*: 230 $[M + H]^+$.

***tert*-Butyl (6-bromothiazolo[5,4-*b*]pyridin-2-yl)carbamate (3).** To a solution of **2** (1.2 g, 5.216 mmol) in THF (26 mL) was added di-*tert*-butyl dicarbonate (1.44 ml, 6.26 mmol), DMAP (64 mg, 0.52 mmol) under nitrogen atmosphere at 0 °C. The mixture was stirred at room temperature for 1 h. The solvent was evaporated and extracted with EtOAc. The organic layer was washed with brine, dried over Na_2SO_4, filtered, and concentrated. The resulting residue was subjected to flash column chromatography on silica gel (10–30% THF/hexane) to afford **3** (1.55 g, 90%) as brown solid. ^{1}H NMR (400 MHz, DMSO-d_6) δ 12.14 (s, 1H), 8.50 (d, J = 2 Hz, 1H), 8.32 (d, J = 2 Hz, 1H), 1.52 (s, 9H); ^{13}C NMR (100 MHz, DMSO-d_6) δ 160.40, 153.37, 152.31, 144.45, 143.58, 128.70, 116.92, 82.00, 27.35; LRMS (ESI) *m/z*: 330 $[M + H]^+$.

***tert*-Butyl (6-(2-methyl-5-nitrophenyl)thiazolo[5,4-*b*]pyridin-2-yl)carbamate (4).** To solution of **3** (1.2 g, 3.62 mmol) and 2-nitrophenylboronic acid pinacol ester (1.1 g, 4.18 mmol) in DME:H_2O (3:1, 0.25 M) was added Pd(dppf)Cl_2 (532 mg, 0.727 mmol), Na_2CO_3 (3.46 g, 32.7 mmol), and purged with nitrogen gas. The mixture was stirred at 100 °C for 3 h, quenched with water, and extracted by CH_2Cl_2. The organic layer was washed with brine, dried over $MgSO_4$, filtered, and concentrated. The resulting residue was subjected to flash column chromatography on silica gel (10–30% THF/hexane) to afford **4** (980 mg, 70%) as pale brown solid. ^{1}H NMR (400 MHz, DMSO-d_6) δ 12.07 (s, 1H), 8.45 (d, J = 2.0 Hz, 1H), 8.21 (dd, J = 8.4 Hz, 2.5 Hz, 1H), 8.14–8.13 (m, 2H), 7.66 (d, J = 8.4 Hz, 1H), 2.38 (s, 3H), 1.53 (s, 9H); ^{13}C NMR (100 MHz, DMSO-d_6) δ 160.21, 154.86, 153.06, 146.07, 144.76, 144.59, 142.59, 139.15, 132.99, 132.00, 127.29, 124.84, 122.91, 82.44, 28.04, 20.62; LRMS (ESI) *m/z*: 387 $[M + H]^+$.

***tert*-Butyl (6-(5-amino-2-methylphenyl)thiazolo[5,4-*b*]pyridin-2-yl)carbamate (5).** To solution of **4** (980 mg, 2.536 mmol) in THF:MeOH:H_2O (4:2:1) was added iron powder (1.42 g, 25.36 mmol), NH_4Cl (2.71 g, 50.72 mmol). The mixture was stirred at 70 °C for 1 h, quenched with water, and extracted by CH_2Cl_2. The organic layer was washed with brine, dried over $MgSO_4$, filtered, and concentrated. The resulting residue was purified by silica gel column chromatography (30–50% THF/hexane) to afford **5** (720 mg, 80%) as white solid. ^{1}H NMR (400 MHz, DMSO-d_6) δ 11.98 (s, 1H), 8.31 (d, J = 2.0 Hz, 1H), 7.91 (d, J = 2.0 Hz, 1H), 6.97 (d, J = 8.4 Hz, 1H), 6.55 (dd, J = 8.4 Hz, 2.5 Hz, 1H), 6.50 (d, J = 2.5 Hz, 1H), 4.98 (s, 2H), 2.07 (s, 3H), 1.53 (s, 9H); ^{13}C NMR (100 MHz, DMSO-d_6) δ 159.56, 153.30, 152.75, 146.67, 144.72, 142.24, 137.73, 135.78, 130.95, 126.65, 121.77, 115.57, 113.85, 82.16, 27.82, 19.02; LRMS (ESI) *m/z*: 357.15 $[M + H]^+$.

General procedure A for the synthesis of 6a–6w. To a solution of **5** (1 equiv) in DMF (0.1 M) was added different carboxylic acids (1.5 equiv), HATU (2 equiv), and DIPEA (3 equiv). The mixture was stirred for 12 h at room temperature, evaporated, diluted with water, and extracted with CH_2Cl_2. The organic layer was washed with brine, dried over $MgSO_4$, filtered, and concentrated. The residue was diluted with CH_2Cl_2, and slowly added TFA at 0 °C. The mixture was stirred at room temperature for 6 h, quenched with saturated sodium carbonate solution, and extracted with CH_2Cl_2. The organic layer was washed with brine, dried over $MgSO_4$, filtered, and concentrated. The resulting crude product was subjected to flash column chromatography on silica gel.

***N*-(3-(2-aminothiazolo[5,4-*b*]pyridin-6-yl)-4-methylphenyl)thiazole-4-carboxamide (6a).** Compound **5** (70 mg, 0.20 mmol) was converted to the target compound using general procedure A. The resulting residue was subjected to flash column chromatography on silica gel (10% MeOH/CH_2Cl_2) to afford **6a** (41 mg, 57%, over 2 steps) as a white solid. ^{1}H NMR (400 MHz, DMSO-d_6) δ 10.34 (bs, 1H), 9.26 (d, J = 2.0 Hz, 1H), 8.48 (d, J = 2.0 Hz, 1H), 8.07 (d, J = 2.0 Hz, 1H), 7.87 (bs, 2H), 7.81 (dd, J = 8.4, 2.5 Hz, 1H), 7.78 (d, J = 2.5 Hz, 1H), 7.59 (d, J = 2.0 Hz, 1H), 7.30 (d, J = 8.4 Hz, 1H), 2.23 (bs, 3H); ^{13}C NMR (100 MHz, DMSO-d_6) δ 166.17, 159.06, 155.02, 154.03, 150.68, 146.52, 141.49, 138.01, 136.49, 134.45, 130.66, 130.62, 125.42, 123.53, 121.79, 119.79, 19.57; LRMS (ESI) *m/z*: 368 [M + H]$^+$.

***N*-(3-(2-aminothiazolo[5,4-*b*]pyridin-6-yl)-4-methylphenyl)-5-methylisoxazole-3-carboxamide (6b).** Compound **5** (70 mg, 0.20 mmol) was converted to the target compound using general procedure A. The resulting residue was subjected to flash column chromatography on silica gel (10% MeOH/CH_2Cl_2) to afford **6b** (45 mg, 63%, over 2 steps) as a white solid. ^{1}H NMR (400 MHz, $CDCl_3$) δ 8.57 (bs, 1H), 8.23 (d, J = 2.0 Hz, 1H), 7.68 (d, J = 2.0 Hz, 1H), 7.63 (dd, J = 8.4, 2.5 Hz, 1H), 7.55 (d, J = 2.5 Hz, 1H), 7.31 (d, J = 8.4 Hz, 1H), 6.53 (bs, 1H), 5.40 (bs, 2H), 2.52 (bs, 3H), 2.27 (bs, 3H); ^{13}C NMR (100 MHz, DMSO-d_6) δ 171.37, 168.20, 159.21, 157.35, 154.11, 146.52, 141.38, 138.12, 136.08, 134.32, 131.20, 130.68, 123.44, 121.85, 119.87, 101.57, 19.57, 11.85; LRMS (ESI) *m/z*: 366 [M + H]$^+$.

***N*-(3-(2-aminothiazolo[5,4-*b*]pyridin-6-yl)-4-methylphenyl)-2-(pyridin-4-yl)thiazole-4-carboxamide (6c).** Compound **5** (70 mg, 0.20 mmol) was converted to the target compound using general procedure A. The resulting residue was subjected to flash column chromatography on silica gel (10% MeOH/CH_2Cl_2) to afford **6c** (36 mg, 42%, over 2 steps) as a white solid. ^{1}H NMR (400 MHz, DMSO-d_6) δ 10.32 (bs, 1H), 8.77 (d, J = 4.6 Hz , 1H), 8.62 (d, J = 1.3 Hz , 1H), 8.11 (m, 3H), 7.88 (bs, 3H), 7.77 (bs, 1H), 7.62 (bs, 1H), 7.34 (d, J = 8.4 Hz, 1H), 2.25 (bs, 3H); ^{13}C NMR (100 MHz, DMSO-d_6) δ 166.63, 165.11, 159.12, 154.53, 151.26, 151.16, 146.96, 141.88, 139.35, 138.58, 136.72, 134.79, 131.36, 131.12, 127.55, 123.94, 122.33, 120.87, 120.22, 20.00; LRMS (ESI) *m/z*: 445 [M + H]$^+$.

***N*-(3-(2-aminothiazolo[5,4-*b*]pyridin-6-yl)-4-methylphenyl)benzamide (6d).** Compound **5** (70 mg, 0.20 mmol) was converted to the target compound using general procedure A. The resulting residue was subjected to flash column chromatography on silica gel (10% MeOH/CH_2Cl_2) to afford **6d** (29 mg, 41%, over 2 steps) as a white solid. ^{1}H NMR (400 MHz, DMSO-d_6) δ 10.26 (bs, 1H), 8.07 (d, J = 2.0 Hz , 1H), 7.96–7.94 (m, 2H), 7.87 (bs, 2H), 7.75 (dd, J = 8.4, 2.5 Hz, 1H), 7.70 (d, J = 2.5 Hz, 1H), 7.59–7.51 (m, 4H), 7.30 (d, J = 8.4 Hz , 1H), 2.23 (bs, 3H); ^{13}C NMR (100 MHz,

DMSO-d_6) 166.18, 165.41, 154.05, 146.53, 141.42, 138.02, 137.17, 134.89, 134.50, 131.52, 130.61, 130.41, 128.37, 127.58, 123.46, 121.70, 119.69, 19.54; LRMS (ESI) *m/z*: 361 $[M + H]^+$.

***N*-(3-(2-aminothiazolo[5,4-*b*]pyridin-6-yl)-4-methylphenyl)-3-methylbenzamide (6e).** Compound **5** (70 mg, 0.20 mmol) was converted to the target compound using general procedure A. The resulting residue was subjected to flash column chromatography on silica gel (10% MeOH/CH_2Cl_2) to afford **7e** (33 mg, 45%, over 2 steps) as a white solid. ^{1}H NMR (400 MHz, DMSO-d_6) δ 10.21 (bs, 1H), 8.07 (d, J = 2 Hz , 1H), 7.87 (bs, 2H), 7.77–7.73 (m, 3H), 7.69 (d, J = 2.2 Hz, 1H), 7.59 (d, J = 2 Hz, 1H), 7.41–7.40 (m, 2H), 7.30 (d, J = 8.4 Hz, 1H), 2.39(bs, 3H), 2.23 (bs, 3H); ^{13}C NMR (100 MHz, MeOD) δ 169.66, 169.26, 155.01, 147.91, 143.40 , 139.80, 139.74, 138.15, 137.07 , 136.39 , 133.69, 133.15 , 132.13 , 129.69, 129.28 , 125.89 , 125.80 , 123.86 , 122.19, 21.54, 20.14; LRMS (ESI) *m/z*: 375 $[M + H]^+$.

***N*-(3-(2-aminothiazolo[5,4-*b*]pyridin-6-yl)-4-methylphenyl)-5-methylnicotinamide (6f).** Compound **5** (70 mg, 0.20 mmol) was converted to the target compound using general procedure A. The resulting residue was subjected to flash column chromatography on silica gel (10% MeOH/CH_2Cl_2) to afford **6f** (26 mg, 36%, over 2 steps) as a white solid. ^{1}H NMR (400 MHz, DMSO-d_6) δ 10.41 (bs, 1H), 8.91 (d, J = 2.0 Hz, 1H), 8.60 (d, J = 2.0 Hz , 1H), 8.11 (s, 1H), 8.07 (d, J = 2.0 Hz, 1H), 7.87 (bs, 2H), 7.74 (dd, J = 8.4, 2.5 Hz , 1H), 7.68 (d, J = 2.5 Hz , 1H), 7.59 (d, J = 2.0 Hz , 1H), 7.32 (d, J = 2.0 Hz , 1H), 2.39 (bs, 3H), 2.23 (bs, 3H); ^{13}C NMR (100 MHz, DMSO-d_6) δ 166.40, 164.24, 154.30, 152.58, 146.74, 146.07, 141.60, 138.31, 137.08, 135.71, 134.63, 133.04, 130.96, 130.90, 130.27, 123.65, 121.88, 119.88, 19.78, 17.98; LRMS (ESI) *m/z*: 376 $[M + H]^+$.

***N*-(3-(2-aminothiazolo[5,4-*b*]pyridin-6-yl)-4-methylphenyl)-3-(methylsulfonyl)benzamide (6g).** Compound **5** (70 mg, 0.20 mmol) was converted to the target compound using general procedure A. The resulting residue was subjected to flash column chromatography on silica gel (10% MeOH/CH_2Cl_2) to afford **6g** (48 mg, 56%, over 2 steps) as a white solid. ^{1}H NMR (400 MHz, DMSO-d_6) δ 10.53 (s, 1H), 8.49 (s, 1H), 8.29 (d, J = 8.0 Hz, 1H), 8.14 (d, J = 8.0 Hz, 1H), 8.08 (d, J = 2.0 Hz, 1H), 7.87 (s, 2H), 7.82 (t, J = 8.0 Hz, 1H), 7.77 (dd, J = 8.4, 2.5 Hz, 1H), 7.69 (d, J = 2.5 Hz, 1 H), 7.60 (d, J = 2.0 Hz, 1H), 7.34 (d, J = 8.4 Hz, 1H), 3.29 (s, 3H), 2.24 (s, 3H); ^{13}C NMR (100 MHz, DMSO-d_6) 166.19, 163.82, 154.11, 146.52, 141.39, 141.12, 138.12, 136.75, 135.84, 134.38, 132.62, 130.91, 130.69, 129.77, 129.75, 126.02, 123.44, 121.88, 119.89, 43.45, 19.55; LRMS (ESI) *m/z*: 439 $[M + H]^+$.

***N*-(3-(2-aminothiazolo[5,4-*b*]pyridin-6-yl)-4-methylphenyl)-3-(trifluoromethyl)benzamide (6h).** Compound **5** (70 mg, 0.20 mmol) was converted to the target compound using general procedure A. The resulting residue was subjected to flash column chromatography on silica gel (10% MeOH/CH_2Cl_2) to afford **6h** (50 mg, 60%, over 2 steps) as a white solid. ^{1}H NMR (400 MHz, DMSO-d_6) δ 10.48 (s, 1H), 8.30 (s, 1H), 8.26 (d, J = 8.0 Hz, 1H), 8.08 (d, J = 2 Hz , 1H), 7.97 (d, J = 8.0 Hz, 1H), 7.88 (bs, 2H), 7.80 (t, J = 8.0 Hz, 1H), 7.76 (dd, J = 8.4, 2.5 Hz, 1H), 7.68 (d, J = 2.5 Hz, 1H), 7.60 (d, J = 2 Hz , 1H), 7.33 (d, J = 8.4 Hz , 1H), 2.23 (bs, 3H); ^{13}C NMR (100 MHz, MeOD) δ 166.69, 164.35, 154.60, 147.03, 141.89, 138.61, 137.28, 136.20, 134.90, 132.27, 131.36, 130.20, 129.61,129.49, 124.66, 124.62, 123.94, 123.09, 122.38. 120.36, 20.04; LRMS (ESI) *m/z*: 429 $[M + H]^+$.

***N*-(3-(2-aminothiazolo[5,4-*b*]pyridin-6-yl)-4-methylphenyl)-2-(3-(trifluoromethyl)phenyl)acetamide (6i).** Compound **5** (70 mg, 0.20 mmol) was converted to the target compound using general procedure A. The resulting residue was subjected to flash column chromatography on silica gel (10% MeOH/CH_2Cl_2) to afford **6i** (40 mg, 46%, over 2 steps) as a white solid. ^{1}H NMR (400 MHz, DMSO-d_6) δ 10.25 (s, 1H), 8.02 (d, J = 2.0 Hz, 1H), 7.86 (s, 2H), 7.69 (s, 1H), 7.60–7.66 (m, 2H), 7.57 (d, J = 8.0 Hz, 1H), 7.49–7.55 (m, 3H), 7.25 (d, J = 8.4 Hz, 1H), 3.77 (s, 2H), 2.18 (s, 3H); ^{13}C NMR (100 MHz, MeOD) δ 171.50, 169.67, 155.01, 147.88, 143.33, 139.80, 138.30, 138.00, 136.99, 134.19, 132.98, 132.20, 130.47, 127.15, 127.11, 125.74, 124.91, 124.88, 122.86, 121.20, 44.26, 20.08; LRMS (ESI) *m/z*: 443 [M + H]$^+$.

1-(3-(2-Aminothiazolo[5,4-*b*]pyridin-6-yl)-4-methylphenyl)-3-(3-(trifluoromethyl)phenyl)urea (6j). To a solution of compound **5** (70 mg, 0.20 mmol) in THF was added 3-(trifluoromethyl)phenyl isocyanate (1.5 equiv) and TEA (3 equiv). The mixture was stirred at room temperature, evaporated, diluted with water, and extracted with CH_2Cl_2. The organic layer was washed with brine, dried over $MgSO_4$, filtered, and concentrated. The residue was diluted with CH_2Cl_2 and slowly added TFA at 0 °C. The mixture was stirred at room temperature for 6 h, quenched with saturated sodium carbonate solution, and extracted with CH_2Cl_2. The organic layer was washed with brine, dried over $MgSO_4$, filtered, and concentrated. The resulting crude product was subjected to flash column chromatography on silica gel to afford **6j** (46 mg, 53%, over 2 steps) as a white solid. ^{1}H NMR (400 MHz, DMSO-d_6) δ 9.06 (bs, 1H), 8.80 (bs, 1H), 8.05 (d, J = 2 Hz, 1H), 8.01 (bs, 1H), 7.86 (bs, 2H), 7.57 (d, J = 2.0 Hz, 1H), 7.55 (bs, 1H), 7.50 (t, J = 8.0 Hz, 1H), 7.41–7.39 (m, 2H), 7.29 (d, J = 8.0 Hz, 1H), 7.24 (d, J = 8.4 Hz, 1H), 2.19 (s, 3H); ^{13}C NMR (100 MHz, DMSO-d_6) 165.73, 153.60, 152.12, 146.09, 141.00, 140.18, 137.81, 136.88, 134.16, 130.36, 129.44, 129.23, 128.47, 123.05, 122.42, 121.39, 119.53, 117.66, 113.70, 18.97; LRMS (ESI) *m/z*: 444 [M + H]$^+$. HRMS (ESI) *m/z* calculated for $C_{21}H_{16}F_3N_5OS$ [M+H]$^+$: 444.11. Found: 444.1109.

***N*-(3-(2-aminothiazolo[5,4-*b*]pyridin-6-yl)-4-methylphenyl)-4-(dimethylamino)-3(trifluoromethyl)benzamide (6k).** Compound **5** (70 mg, 0.20 mmol) was converted to the target compound using general procedure A. The resulting residue was subjected to flash column chromatography on silica gel (10% MeOH/CH_2Cl_2) to afford **6k** (67 mg, 73%, over 2 steps) as a white solid. ^{1}H NMR (400 MHz, DMSO-d_6) δ 10.28 (bs, 1H), 8.23 (d, J = 2.0 Hz , 1H), 8.16 (dd, J = 8.0 Hz, 2.0 Hz, 1H), 8.07 (d, J = 2 Hz, 1H), 7.87 (bs, 2H), 7.75 (dd, J = 8.4 Hz, 2.5 Hz, 1H), 7.66 (d, J = 2.5 Hz, 1H), 7.59 (d, J = 2.0 Hz, 1H), 7.45 (d, J = 8.8 Hz , 1H), 7.31 (d, J = 8.4 Hz , 1H), 2.81 (bs, 6H), 2.23 (bs, 3H); ^{13}C NMR (100 MHz, MeOD) δ 169.66, 167.27, 157.17, 155.01, 147.90, 143.39, 139.74, 138.04, 137.04, 133.21, 132.14, 129.18, 129.13, 127.15, 125.79, 123.92, 123.80, 123.50, 122.42, 122.25, 45.22, 45.19, 20.14; LRMS (ESI) *m/z*: 472 [M + H]$^+$.

***N*-(3-(2-aminothiazolo[5,4-*b*]pyridin-6-yl)-4-methylphenyl)-4-morpholino-3-(trifluoromethyl)benzamide (6l).** Compound **5** (70 mg, 0.20 mmol) was converted to the target compound using general procedure A. The resulting residue was subjected to flash column chromatography on silica gel (10% MeOH/CH_2Cl_2) to afford **6l** (50 mg, 50%, over 2 steps) as a white solid. ^{1}H NMR (400 MHz, DMSO-d_6) δ 10.38 (bs, 1H), 8.24 (d, J = 8.0 Hz, 2H), 8.07 (d, J = 2 Hz, 1H), 7.88 (bs, 2H), 7.75 (dd, J = 8.4 Hz, 2.5 Hz, 1H), 7.67 (d, J = 2.5 Hz, 1H) 7.64 (s, 1H), 7.59 (d, J = 2 Hz , 1H), 7.32 (d, J = 8.4 Hz, 1H), 3.73 (t, J = 4.8 Hz, 4H), 2.96 (t, J = 4.8 Hz, 4H), 2.23 (bs, 3H); ^{13}C NMR (100 MHz, MeOD)

169.65, 167.01, 156.51, 155.02, 147.90, 143.38, 139.76, 137.94, 136.98, 133.69, 133.34, 132.39, 132.17, 128.49, 128.43, 127.63, 127.34, 125.77, 125.01, 123.89, 68.37, 54.84, 20.15; LRMS (ESI) *m/z*: 514 $[M + H]^+$.

***N*-(3-(2-aminothiazolo[5,4-*b*]pyridin-6-yl)-4-methylphenyl)-4-(4-methylpiperazin-1-yl)-3-(trifluoromethyl)benzamide (6m).** Compound **5** (70 mg, 0.20 mmol) was converted to the target compound using general procedure A. The resulting residue was subjected to flash column chromatography on silica gel (10% $MeOH/CH_2Cl_2$) to afford **6m** (59 mg, 57%, over 2 steps) as a white solid. ^{1}H NMR (400 MHz, $CDCl_3$) δ 8.24 (d, J = 2 Hz, 1H), 8.14 (d, J = 2 Hz , 1H), 8.03 (d, J = 8.0 Hz, 1H), 7.78 (bs, 1H), 7.65 (dd, J = 8.4 Hz, 2.5 Hz, 1H), 7.50 (d, J = 2.5 Hz , 1H), 7.42 (d, J = 8.0 Hz, 1H), 7.33 (d, J = 8.4 Hz , 1H), 5.34 (bs, 2H), 3.10 (bs, 4H), 2.44 (bs, 3H), 2.28 (bs, 3H), 1.27 (d, J = 4.7 Hz, 4H); ^{13}C NMR (100 MHz, MeOD) δ 169.65, 167.01, 156.48, 155.03, 147.91, 143.38, 139.77, 137.95, 137.00. 133.62, 133.35, 132.47, 132.18, 128.49, 128.44, 127.37, 125.77, 125.07, 123.89, 122.21, 56.32, 53.84, 46.15, 14.57; LRMS (ESI) *m/z*: 527 $[M + H]^+$.

***N*-(3-(2-aminothiazolo[5,4-*b*]pyridin-6-yl)-4-methylphenyl)-3-morpholino-5-(trifluoromethyl)benzamide (6n).** Compound **5** (70 mg, 0.20 mmol) was converted to the target compound using general procedure A. The resulting residue was subjected to flash column chromatography on silica gel (10% $MeOH/CH_2Cl_2$) to afford **6n** (55 mg, 55%, over 2 steps) as a white solid. ^{1}H NMR (400 MHz, DMSO-d_6) δ 10.36 (bs, 1H), 8.07 (d, J = 2.0 Hz , 1H), 7.87 (bs, 2H), 7.75 (dd, J = 8.4 Hz, 2.5 Hz, 1H), 7.71 (bs, 1H), 7.65 (d, J = 2.5 Hz, 2H), 7.59 (d, J = 2.0 Hz , 1H), 7.39 (bs, 1H), 7.32 (d, J = 8.4 Hz , 1H), 3.77 (t, J = 4.8 Hz, 4H), 3.29 (t, J = 4.8 Hz, 4H), 2.23 (bs, 3H); ^{13}C NMR (100 MHz, Acetone-d_6) δ 167.31, 165.38, 155.91, 153.05, 147.79, 143.23, 139.70, 138.28, 138.16, 136.04, 132.17, 131.74, 125.22, 122.85, 120.81, 118.25, 114.95, 114.91, 114.62, 114.59, 67.24, 49.21, 20.08; LRMS (ESI) *m/z*: 514 $[M + H]^+$.

***N*-(3-(2-aminothiazolo[5,4-*b*]pyridin-6-yl)-4-methylphenyl)-3-(4-methylpiperazin-1-yl)-5-(trifluoromethyl)benzamide (6o).** Compound **5** (70 mg, 0.20 mmol) was converted to the target compound using general procedure A. The resulting residue was subjected to flash column chromatography on silica gel (10% $MeOH/CH_2Cl_2$) to afford **6o** (43 mg, 42%, over 2 steps) as a white solid. ^{1}H NMR (400 MHz, Acetone-d_6) δ 9.69 (s, 1H), 8.12 (d, J = 2 Hz, 1H), 7.80 (dd, J = 8.4 Hz, 2.5 Hz, 1H), 7.78 (s, 1H), 7.72 (d, J = 2.5 Hz, 1H), 7.64 (s, 1H), 7.61 (d, J = 2.0 Hz, 1H), 7.37 (s, 1H), 7.32 (d, J = 8.4 Hz, 1H), 7.13 (bs, 1H), 3.39–3.33 (m, 4H), 2.57–2.52 (m, 4H), 2.28 (d, J = 2.32 Hz, 6H); ^{13}C NMR (100 MHz, DMSO-d_6) δ 166.18, 164.31, 154.08, 151.26, 146.52, 141.39, 138.07, 136.80, 136.55, 134.39, 130.76, 130.62, 130.25, 129.95, 123.43, 122.76, 121.99, 119.96, 117.26, 113.41, 54.28, 47.34, 45.61, 19.53; LRMS (ESI) *m/z*: 527 $[M + H]^+$. HRMS (ESI) *m/z* calculated for $C_{26}H_{25}F_3N_6OS$ $[M+H]^+$: 527.1841. Found: 527.1857.

***N*-(3-(2-aminothiazolo[5,4-*b*]pyridin-6-yl)-4-methylphenyl)-4-(morpholinomethyl)-3-(trifluoromethyl)benzamide (6p).** Compound **5** (21 mg, 0.06 mmol) was converted to the target compound using general procedure A. The resulting residue was subjected to flash column chromatography on silica gel (0–10% $MeOH/CH_2Cl_2$) to afford **6p** (21 mg, 67%, over 2 steps) as a white solid. ^{1}H NMR (300 MHz, DMSO-d_6) δ 10.45 (s, 1H), 8.26 (s, 1H), 8.23 (d, J = 8.4 Hz, 1H), 8.08 (d, J = 1.9 Hz, 1H), 7.95 (d, J = 8.1 Hz, 0H), 7.87 (br s, 2H), 7.75 (dd,

J = 8.3, 2.2 Hz, 1H), 7.68 (d, J = 2.3 Hz, 1H), 7.59 (d, J = 2.0 Hz, 1H), 7.32 (d, J = 8.4 Hz, 1H), 3.69 (s, 2H), 3.61 (t, J = 4.6 Hz, 4H), 2.41 (t, J = 4.6 Hz, 4H), 2.23 (s, 3H); ^{13}C NMR (100 MHz, MeOD) δ 168.07, 165.53, 153.41, 146.33, 141.77, 141.07, 138.18, 136.35, 135.40, 133.89, 131.81, 130.75, 130.69, 130.63, 128.74, 128.43, 125.53, 124.99, 124.94, 124.17, 122.25, 120.57, 66.61, 58.13, 53.42, 18.64; LRMS (ESI) *m/z*: 528 $[M + H]^+$.

***N*-(3-(2-aminothiazolo[5,4-*b*]pyridin-6-yl)-4-methylphenyl)-4-((1,1-dioxidothiomorpholino)methyl)-3-(trifluoromethyl)benzamide (6q).** Compound **5** (20 mg, 0.05 mmol) was converted to the target compound using general procedure A. The resulting residue was subjected to flash column chromatography on silica gel (0–5% MeOH/CH_2Cl_2) to afford **6q** (9 mg, 27%, over 2 steps) as a beige solid. ^{1}H NMR (300 MHz, DMSO-d_6) δ 10.47 (s, 1H), 8.28 (s, 1H), 8.25 (d, J = 8.2 Hz, 1H), 8.09 (d, J = 2.0 Hz, 1H), 8.02 (d, J = 7.9 Hz, 1H), 7.88 (s, 2H), 7.76 (dd, J = 8.4, 2.2 Hz, 1H), 7.69 (d, J = 2.3 Hz, 1H), 7.60 (d, J = 2.0 Hz, 1H), 7.34 (d, J = 8.4 Hz, 1H), 3.90 (s, 2H), 3.25–3.10 (m, 4H), 3.01–2.84 (m, 4H), 2.70 (s, 3H), 2.24 (s, 3H); LRMS (ESI) *m/z*: 576 $[M + H]^+$.

***N*-(3-(2-aminothiazolo[5,4-*b*]pyridin-6-yl)-4-methylphenyl)-4-((4-methylpiperazin-1-yl)methyl)-3-(trifluoromethyl)benzamide (6r).** Compound **5** (70 mg, 0.20 mmol) was converted to the target compound using general procedure A. The resulting residue was subjected to flash column chromatography on silica gel (10% MeOH/CH_2Cl_2) to afford **6r** (70 mg, 65%, over 2 steps) as a white solid. ^{1}H NMR (400 MHz, DMSO-d_6) δ 10.45 (s, 1H), 8.25 (s, 1H), 8.23 (d, J = 8.0 Hz, 1H), 8.07 (d, J = 2.0 Hz, 1H), 7.92 (d, J = 8.0 Hz , 1H), 7.87 (bs, 2H), 7.75 (dd, J = 8.4 Hz, 2.5 Hz, 1H), 7.67 (d, J = 2.5 Hz , 1H), 7.59 (d, J = 2.0 Hz, 1H), 7.32 (d, J = 8.4 Hz, 1H), 3.68 (bs, 2H), 2.43 (m, 8H), 2.23 (bs, 3H), 2.19 (bs, 3H); ^{13}C NMR (100 MHz, MeOD) 169.66, 167.14, 155.05, 147.92, 143.37, 142.78, 139.80, 137.91, 136.98, 135.52, 133.43, 132.27, 132.19, 129.98, 127.11, 126.50, 125.77, 123.87, 122.19, 59.10, 56.07, 53.69, 45.93, 20.16; LRMS (ESI) *m/z*: 541 $[M + H]^+$. HRMS (ESI) *m/z* calculated for $C_{27}H_{27}F_3N_6OS$ $[M+H]^+$: 541.20. Found: 541.2014.

***N*-(3-(2-aminothiazolo[5,4-*b*]pyridin-6-yl)-4-methylphenyl)-4-(piperazin-1-ylmethyl)-3-(trifluoromethyl)benzamide (6s).** Compound **5** (70 mg, 0.20 mmol) was converted to the target compound using general procedure A. The resulting residue was subjected to flash column chromatography on silica gel (10% MeOH/CH_2Cl_2) to afford **6s** (44 mg, 43%, over 2 steps) as a white solid. ^{1}H NMR (400 MHz, DMSO-d_6) δ 10.44 (s, 1H), 8.24 (s, 1H), 8.22 (d, J = 8.0 Hz, 1H), 8.07 (d, J = 2 Hz, 1H), 7.94 (d, J = 8.0 Hz, 1H), 7.87 (s, 2H), 7.75 (dd, J = 8.4 Hz, 2.5 Hz, 1H), 7.68 (d, J = 2.5 Hz, 1H), 7.59 (d, J = 2 Hz, 1H), 7.32 (d, J = 8.4 Hz, 1H), 3.65 (s, 2H), 2.74 (m, 4H), 2.35 (m, 4H), 2.23 (bs, 3H); ^{13}C NMR (100 MHz, MeOD) δ 169.66, 167.14, 155.04, 147.91, 143.37, 142.78, 139.80, 137.91, 136.98, 135.49, 133.43, 132.28, 132.19, 130.30, 130.00, 126.56, 126.50, 125.77, 123.87, 122.19, 59.73, 54.57, 46.41, 20.17; LRMS (ESI) *m/z*: 527 $[M + H]^+$. HRMS (ESI) *m/z* calculated for $C_{26}H_{25}F_3N_6OS$ $[M+H]^+$: 527.18. Found: 527.1847.

***N*-(3-(2-aminothiazolo[5,4-*b*]pyridin-6-yl)-4-methylphenyl)-4-((4-(2-hydroxyethyl)piperazin-1-yl)methyl)-3-(trifluoromethyl)benzamide (6t).** Compound **5** (30 mg, 0.09 mmol) was converted to the target compound using general procedure A. The resulting residue was subjected to flash column chromatography on silica gel (0–15%

MeOH/CH_2Cl_2) to afford **6t** (22 mg, 45%, over 2 steps) as a beige solid. ^{1}H NMR (300 MHz, DMSO-d_6) δ 10.46 (s, 1H), 8.31–8.20 (m, 2H), 8.08 (d, J = 2.0 Hz, 1H), 7.93 (d, J = 8.2 Hz, 1H), 7.88 (s, 2H), 7.76 (dd, J = 8.3, 2.3 Hz, 1H), 7.69 (d, J = 2.3 Hz, 1H), 7.60 (d, J = 2.0 Hz, 1H), 7.33 (d, J = 8.4 Hz, 1H), 4.53–4.35 (m, 1H), 3.68 (s, 2H), 3.50 (q, J = 5.9 Hz, 2H), 2.44 (br s, 9H), 2.24 (s, 3H); ^{13}C NMR (75 MHz, MeOD) δ 168.07, 165.53, 153.46, 146.34, 141.80, 141.30, 138.20, 136.35, 135.39, 133.87, 131.82, 130.63, 124.99, 124.91, 124.20, 122.28, 120.59, 59.77, 58.18, 53.11, 52.28, 18.65; LRMS (ESI) *m/z*: 571 $[M + H]^+$.

4-((4-Acetylpiperazin-1-yl)methyl)-*N*-(3-(2-aminothiazolo[5,4-*b*]pyridin-6-yl)-4-methylphenyl)-3-(trifluoromethyl)benzamide (6u). Compound **5** (80 mg, 0.22 mmol) was converted to the target compound using general procedure A. The resulting residue was subjected to flash column chromatography on silica gel (0–7.5% MeOH/CH_2Cl_2) to afford **6u** (88 mg, 70%, over 2 steps) as a beige solid. ^{1}H NMR (300 MHz, DMSO-d_6) δ 10.47 (s, 1H), 8.38–8.17 (m, 2H), 8.09 (d, J = 1.9 Hz, 1H), 7.98 (d, J = 8.0 Hz, 1H), 7.88 (s, 2H), 7.76 (dd, J = 8.3, 2.2 Hz, 1H), 7.69 (d, J = 2.2 Hz, 1H), 7.60 (d, J = 1.9 Hz, 1H), 7.34 (d, J = 8.4 Hz, 1H), 3.72 (s, 2H), 3.47 (q, J = 4.8 Hz, 4H), 2.43 (t, J = 5.0 Hz, 2H), 2.37 (t, J = 5.0 Hz, 2H), 2.25 (s, 3H), 2.00 (s, 3H); ^{13}C NMR (100 MHz, DMSO-d_6) δ 168.62, 166.65, 164.32, 154.55, 147.00, 141.86, 141.22, 138.56, 137.32, 134.88, 134.33, 132.06, 131.26, 131.23, 131.16, 127.89, 127.58, 125.94, 125.47, 123.92, 123.22, 122.27, 120.27, 57.80, 53.42, 52.95, 46.10, 41.29, 31.16, 21.66, 20.06; LRMS (ESI) *m/z*: 569 $[M + H]^+$.

***N*-(3-(2-aminothiazolo[5,4-*b*]pyridin-6-yl)-4-methylphenyl)-4-((3-(dimethylamino)piperidin-1-yl)methyl)-3-(trifluoromethyl)benzamide (6v).** Compound **5** (100 mg, 0.28 mmol) was converted to the target compound using general procedure A. The resulting residue was subjected to flash column chromatography on silica gel (0–20% MeOH/CH_2Cl_2) to afford **6v** (83 mg, 52%, over 2 steps) as a beige solid. ^{1}H NMR (400 MHz, MeOD) δ 8.22 (d, J = 1.9 Hz, 1H), 8.14 (dd, J = 8.2, 1.9 Hz, 1H), 8.07 (d, J = 1.9 Hz, 1H), 7.96 (d, J = 8.2 Hz, 1H), 7.66–7.61 (m, 2H), 7.59 (d, J = 1.9 Hz, 1H), 7.26 (d, J = 8.1 Hz, 1H), 3.73 (s, 2H), 3.01–2.92 (m, 1H), 2.89–2.79 (m, 1H), 2.68–2.61 (m, 1H), 2.33–2.24 (m, 1H), 2.21–2.08 (m, 1H), 2.06–1.89 (m, 1H), 1.84–1.70 (m, 1H), 1.68–1.54 (m, 1H), 1.53–1.40 (m, 1H); ^{13}C NMR (100 MHz, MeOD) δ 166.40, 163.78, 151.82, 144.70, 140.16, 139.79, 136.52, 134.74, 133.70, 132.20, 130.13, 129.19, 129.04, 128.96, 126.91, 126.61, 123.92, 123.37, 123.32, 122.55, 121.20, 120.63, 118.93, 60.33, 56.24, 53.13, 51.57, 38.87, 23.63, 21.51, 17.12; LRMS (ESI) *m/z*: 569 $[M + H]^+$.

***N*-(3-(2-aminothiazolo[5,4-*b*]pyridin-6-yl)-4-methylphenyl)-4-(((2-(dimethylamino)ethyl)(methyl)amino)methyl)-3-(trifluoromethyl)benzamide (6w).** Compound **5** (100 mg, 0.28 mmol) was converted to the target compound using general procedure A. The resulting residue was subjected to flash column chromatography on silica gel (0–20% MeOH/CH_2Cl_2) to afford **6w** (63 mg, 42%, over 2 steps) as a white solid. ^{1}H NMR (400 MHz, DMSO-d_6) δ 10.48 (s, 1H), 8.25 (s, 1H), 8.24 (d, J = 10.4 Hz, 2H), 8.08 (d, J = 2.0 Hz, 1H), 7.98 (d, J = 8.0 Hz, 1H), 7.91 (s, 2H), 7.76 (dd, J = 8.3, 2.3 Hz, 1H), 7.68 (d, J = 2.2 Hz, 1H), 7.60 (d, J = 2.0 Hz, 1H), 7.33 (d, J = 8.4 Hz, 1H), 3.72 (s, 2H), 2.55 (s, 4H), 2.27 (s, 6H), 2.23 (s, 3H), 2.18 (s, 3H); ^{13}C NMR (75 MHz, MeOD) δ 168.05, 165.49, 153.47, 146.33, 141.98, 141.79, 138.19, 136.34, 135.36, 133.86, 131.81, 130.80, 130.75, 130.63, 128.57,

128.16, 124.92, 124.84, 124.19, 122.30, 120.60, 57.83, 56.20, 54.27, 43.94, 41.35, 18.66; LRMS (ESI) *m/z*: 543 [M + H]$^+$.

General Procedure B for the synthesis of compound 7a–h. To solution of a substrate (1 equiv) in CH_2Cl_2 was added acetic anhydride or different acyl chloride (1.2 equiv), and pyridine (3 equiv). The mixture was stirred for 3–6 h at room temperature, evaporated then diluted with water, and extracted with CH_2Cl_2. The organic layer was washed with brine, dried over $MgSO_4$, filtered, and concentrated. The resulting crude product was subjected to flash column chromatography on silica gel (10% MeOH/CH_2Cl_2) to afford **7a**–**h**.

***N*-(3-(2-(cyclohexanecarboxamido)thiazolo[5,4-*b*]pyridin-6-yl)-4-methylphenyl)-4-((4-methylpiperazin-1-yl)methyl)-3-(trifluoromethyl)benzamide (7a).** Compound **6r** (40 mg, 0.078 mmol) was converted to the target compound using general procedure B. The resulting residue was subjected to flash column chromatography on silica gel (10% MeOH/CH_2Cl_2) to afford **7a** (12 mg, 25%) as a white solid. ^{1}H NMR (400 MHz, DMSO-d_6) δ 12.49 (bs, 1H), 10.47 (s, 1H), 8.45 (d, J = 2.0 Hz, 1H), 8.25 (s, 1H), 8.22 (d, J = 8.0 Hz, 1H), 8.07 (s, 1H), 7.92 (d, J = 8.0 Hz, 1H), 7.77 (dd, J = 8.4, 2.5 Hz, 1H), 7.73 (d, J = 2.5 Hz, 1H), 7.36 (d, J = 8.4 Hz, 1H), 3.67 (s, 2H), 2.41 (bs, 4H), 2.33 (bs, 3H), 2.25 (s, 3H), 2.16 (s, 3H), 2.08 (s, 1H), 1.90–1.86 (m, 2H), 1.77 (d, J = 12.7 Hz, 2H), 1.66 (d, J = 10.5 Hz, 1H), 1.4 (d, J = 11.5 Hz, 2H), 1.30–1.24 (m, 4H); ^{13}C NMR (100 MHz, MeOD) δ 177.74, 167.18, 160.62, 155.51, 146.72, 143.82, 142.77, 139.59, 137.98, 137.20, 135.54, 133.52, 132.30, 132.27, 130.32, 130.01, 129.35, 126.52, 124.05, 122.34, 59.07, 56.04, 53.60, 46.10, 45.85, 30.45, 26.93, 26.71, 20.15; LRMS (ESI) *m/z*: 651 [M + H]$^+$. HRMS (ESI) *m/z* calculated for $C_{34}H_{37}F_3N_6O_2S$ [M+H]$^+$: 651.27. Found: 651.2739.

***N*-(3-(2-benzamidothiazolo[5,4-*b*]pyridin-6-yl)-4-methylphenyl)-4-((4-methylpiperazin-1-yl)methyl)-3-(trifluoromethyl)benzamide (7b).** Compound **6r** (40 mg, 0.078 mmol) was converted to the target compound using general procedure B. The resulting residue was subjected to flash column chromatography on silica gel (10% MeOH/CH_2Cl_2) to afford **7b** (16 mg, 33%) as a white solid. ^{1}H NMR (400 MHz, DMSO-d_6) δ 13.01 (s, 1H), 10.48 (s, 1H), 8.48 (d, J = 2.0 Hz, 1H), 8.26 (s, 1H), 8.23 (d, J = 8.0 Hz, 1H), 8.18–8.15 (m, 2H), 8.11 (d, J = 2.0 Hz, 1H), 7.92 (d, J = 8.0 Hz, 1H), 7.79 (dd, J = 8.4, 2.5 Hz, 1H), 7.74 (d, J = 2.0 Hz, 1H), 7.68 (m, 1H), 7.60–7.56 (m, 2H), 7.37 (d, J = 8.4 Hz, 1H), 3.68 (bs, 2H), 2.43 (m, 8H), 2.27 (s, 3H), 2.20 (s, 3H); ^{13}C NMR (100 MHz, MeOD) δ 168.57, 167.15, 161.36, 155.55, 146.90, 143.88, 142.76, 139.58, 137.99, 137.22, 135.52, 134.37, 133.69, 133.50, 132.29, 132.27, 130.29, 130.08, 129.43, 129.04, 127.11, 126.52, 124.05, 122.33, 59.08, 56.03, 53.60, 45.85, 20.17; LRMS (ESI) *m/z*: 645 [M + H]$^+$. HRMS (ESI) *m/z* calculated for $C_{34}H_{31}F_3N_6O_2S$ [M+H]$^+$: 645.22. Found: 645.227.

***N*-(3-(2-acetamidothiazolo[5,4-*b*]pyridin-6-yl)-4-methylphenyl)-4-((4-methylpiperazin-1-yl)methyl)-3-(trifluoromethyl)benzamide (7c).** Compound **6r** (40 mg, 0.078 mmol) was converted to the target compound using general procedure B. The resulting residue was subjected to flash column chromatography on silica gel (10% MeOH/CH_2Cl_2) to afford **7c** (16 mg, 38%) as a white solid. ^{1}H NMR (400 MHz, DMSO-d_6) δ 12.55 (bs, 1H), 10.47 (s, 1H), 8.46 (d, J = 2.0 Hz, 1H), 8.25 (s, 1H) 8.22 (d, J = 8.0 Hz, 1H), 8.10 (d, J = 2.0 Hz, 1H), 7.92 (d, J = 8.0 Hz, 1H), 7.77 (dd, J = 8.4, 2.5 Hz, 1H), 7.73 (d, J = 2.5 Hz, 1H), 7.36 (d, J = 8.4 Hz, 1H), 3.68 (s, 2H), 2.42–2.33 (m, 8H), 2.25

(d, J = 4.6 Hz, 6H), 2.16 (s, 3H); ^{13}C NMR (100 MHz, MeOD) δ 171.85, 167.20, 160.52, 155.48, 146.78, 143.80, 142.84, 139.58, 137.99, 137.23, 135.52, 133.52, 132.31, 132.28, 130.32, 130.01, 129.43, 126.51, 124.05, 122.36, 59.12, 56.08, 53.71, 45.96, 23.02, 20.15; LRMS (ESI) *m/z*: 583 [M + H]$^+$. HRMS (ESI) *m/z* calculated for $C_{29}H_{29}F_3N_6O_2S$ [M+H]$^+$: 583.21. Found: 583.2111.

***N*-(3-(2-(cyclohexanecarboxamido)thiazolo[5,4-*b*]pyridin-6-yl)-4-methylphenyl)-4-morpholino-3-(trifluoromethyl)benzamide (7d).** Compound **6l** (40 mg, 0.078 mmol) was converted to the target compound using general procedure B. The resulting residue was subjected to flash column chromatography on silica gel (10% MeOH/CH_2Cl_2) to afford **7d** (12 mg, 25%) as a white solid. ^{1}H NMR (400 MHz, DMSO-d_6) δ 12.51 (s, 1H), 10.40 (s, 1H), 8.45 (d, J = 2.0 Hz, 1H), 8.25–8.23 (m, 2H), 8.07 (d, J = 2.0 Hz, 1H), 7.77 (dd, J = 8.4 Hz, 2.5 Hz, 1H), 7.72 (d, J = 2.5 Hz, 1H), 7.65 (d, J = 8.0 Hz, 1H), 7.35 (d, J = 8.4 Hz, 1H), 3.74–3.72 (m, 4H), 2.97–2.95 (m, 4H), 2.25 (s, 3H), 1.88 (d, J = 12.10 Hz, 2H), 1.77 (d, J = 12.23 Hz, 2H), 1.66 (d, J = 10.39 Hz, 1H), 1.48–1.39 (m, 2H), 1.35–1.19 (m, 4H); ^{13}C NMR (100 MHz, MeOH-d_6) δ 177.63, 166.94, 160.52, 156.46, 155.41, 146.66, 143.70, 139.46, 137.97, 137.04, 133.69, 133.30, 132.29, 132.22, 129.28, 128.49, 128.44, 127.25, 124.93, 124.02, 122.22, 68.34, 54.82, 46.07, 30.43, 26.90, 26.70, 20.20; LRMS (ESI) *m/z*: 624 [M + H]$^+$.

***N*-(3-(2-acetamidothiazolo[5,4-*b*]pyridin-6-yl)-4-methylphenyl)-4-morpholino-3-(trifluoromethyl)benzamide (7e).** Compound **6l** (40 mg, 0.078 mmol) was converted to the target compound using general procedure B. The resulting residue was subjected to flash column chromatography on silica gel (10% MeOH/CH_2Cl_2) to afford **7e** (13 mg, 31%) as a white solid. ^{1}H NMR (400 MHz, DMSO-d_6) δ 12.55 (s, 1H), 10.40 (s, 1H), 8.46 (d, J = 2.0 Hz, 1H), 8.25–8.23 (m, 2H), 8.09 (d, J = 2.0 Hz, 1H), 7.77 (dd, J = 8.4 Hz, 2.5 Hz, 1H), 7.72 (d, J = 2.5 Hz, 1H), 7.65 (d, J = 8.0 Hz, 1H), 7.35 (d, J = 8.4 Hz, 1H), 3.74–3.72 (m, 4H), 2.97–2.95 (m, 4H), 2.25 (d, J = 3.3 Hz, 6H); ^{13}C NMR (100 MHz, MeOD) δ 171.76, 166.91, 160.36, 156.45, 155.36, 146.72, 143.66, 139.42, 137.97, 137.06, 133.68, 133.29, 132.27, 132.21, 128.49, 128.43, 127.53, 127.24, 124.91, 123.97, 122.22, 68.34, 54.81, 23.05, 20.18; LRMS (ESI) *m/z*: 556 [M + H]$^+$. HRMS (ESI) *m/z* calculated for $C_{27}H_{24}F_3N_5O_3S$ [M+H]$^+$ 556.16. Found 556.1611.

***N*-(3-(2-acetamidothiazolo[5,4-*b*]pyridin-6-yl)-4-methylphenyl)-4-(morpholinomethyl)-3-(trifluoromethyl)benzamide (7f).** Compound **6p** (26 mg, 0.049 mmol) was converted to the target compound using general procedure B. The resulting residue was subjected to flash column chromatography on silica gel (0–10% MeOH/CH_2Cl_2) to afford **7f** (11 mg, 40%) as a white solid. ^{1}H NMR (300 MHz, DMSO-d_6) δ 12.55 (bs, 1H), 10.49 (s, 1H), 8.47 (d, J = 2.0 Hz, 1H), 8.27 (s, 1H), 8.24 (d, J = 8.7 Hz, 1H), 8.10 (d, J = 1.9 Hz, 1H), 7.96 (d, J = 8.0 Hz, 1H), 7.78 (dd, J = 8.3, 2.2 Hz, 1H), 7.74 (d, J = 2.2 Hz, 1H), 7.37 (d, J = 8.4 Hz, 1H), 3.70 (s, 2H), 3.62 (t, J = 4.6 Hz, 4H), 2.42 (t, J = 4.6 Hz, 4H), 2.26 (d, J = 4.3 Hz, 6H); ^{13}C NMR (100 MHz, Acetone-d_6) δ 169.18, 163.92, 158.31, 154.36, 145.57, 141.88, 141.24, 138.28, 137.22, 135.37, 134.25, 131.23, 131.06, 130.93, 130.85, 128.36, 128.06, 127.25, 125.73, 125.01, 124.96, 123.01, 121.93, 119.97, 66.53, 58.24, 53.63, 22.26, 19.12; LRMS (ESI) *m/z*: 570 [M + H]$^+$.

***N*-(3-(2-acetamidothiazolo[5,4-*b*]pyridin-6-yl)-4-methylphenyl)-4-((3-(dimethylamino)piperidin-1-yl)methyl)-3-(trifluoromethyl)benzamide (7g).** Compound **6v** (19 mg, 0.034 mmol) was converted to the target compound using

general procedure B. The resulting residue was subjected to flash column chromatography on silica gel (0–15% MeOH/CH_2Cl_2) to afford **7g** (14 mg, 68%) as a white solid. 1H NMR (300 MHz, DMSO-d_6) δ 10.48 (s, 1H), 8.47 (d, J = 1.9 Hz, 1H), 8.31–8.16 (m, 2H), 8.11 (d, J = 1.9 Hz, 1H), 7.94 (d, J = 7.9 Hz, 1H), 7.79 (dd, J = 8.1, 2.2 Hz, 1H), 7.75 (d, J = 2.2 Hz, 1H), 7.37 (d, J = 8.3 Hz, 1H), 3.69 (s, 2H), 2.97–2.85 (m, 1H), 2.78–2.61 (m, 1H), 2.26 (s, 3H), 2.25 (s, 3H), 2.18 (s, 6H), 2.03–1.74 (m, 3H), 1.68 (d, J = 13.1Hz, 1H), 1.46 (d, J = 12.5 Hz, 2H); ^{13}C NMR (100 MHz, Acetone) δ 169.18, 163.98, 163.91, 158.30, 154.35, 145.57, 142.16, 141.89, 138.27, 137.26, 137.17, 135.38, 134.07, 134.04, 131.17, 131.03, 130.84, 130.58, 128.10, 127.80, 127.25, 125.77, 124.96, 124.91, 123.05, 121.93, 121.84, 119.97, 119.88, 61.42, 58.23, 56.82, 53.76, 41.24, 26.13, 24.47, 22.26, 19.12; LRMS (ESI) *m/z*: 611 $[M + H]^+$.

***N*-(3-(2-acetamidothiazolo[5,4-*b*]pyridin-6-yl)-4-methylphenyl)-4-(((2-(dimethylamino)ethyl)(methyl)amino)methyl)-3-(trifluoromethyl)benzamide (7h).** Compound **6w** (13 mg, 0.024 mmol) was converted to the target compound using general procedure B. The resulting residue was subjected to flash column chromatography on silica gel (0–20% MeOH/CH_2Cl_2) to afford **7h** (13 mg, 92%) as a white solid. ^{1}H NMR (300 MHz, MeOD) δ 8.44 (d, J = 1.9 Hz, 1H), 8.29 (s, 1H), 8.20 (dd, J = 8.1, 1.8 Hz, 1H), 8.12–7.97 (m, 3H), 7.74–7.64 (m, 2H), 7.36 (d, J = 8.1Hz, 1H), 6.78 (d, J = 6.6 Hz, 1H), 3.81 (s, 2H), 2.83 (t, J = 6.7 Hz, 2H), 2.69 (t, J = 6.6 Hz, 2H), 2.49 (s, 6H), 2.31 (s, 3H), 2.29 (s, 6H); ^{13}C NMR (100 MHz, MeOD) δ 163.89, 157.31, 152.24, 143.57, 140.59, 140.15, 136.36, 134.81, 134.00, 132.66, 132.34, 130.38, 130.32, 129.32, 129.13, 126.26, 123.94, 120.84, 119.13, 105.02, 56.22, 53.99, 51.78, 47.69, 41.83, 39.46, 36.68, 27.70, 19.91, 17.06; LRMS (ESI) *m/z*: 585 $[M + H]^+$.
