# Supplementary material for: Identification of Thiazolo[5,4-b]pyridine Derivatives as c-KIT Inhibitors for Overcoming Imatinib Resistance
Source: Cancers (Basel). 2022 Dec 26;15(1):143. doi: 10.3390/cancers15010143 (PMC9817970; doi:10.3390/cancers15010143)
Supplement: Supplementary file 1 [file cancers-15-00143-s001.zip › Table S1_c-KIT_tbsim_12-26-2022.pdf]

Supplementary Materials

Identification of Thiazolo[5,4-*b*]pyridine Derivatives as c-KIT Inhibitors for Overcoming Imatinib Resistance

Yunju Nam[1,2,3,†], Chan Kim[2,†], Junghee Han[1,2,3], SeongShick Ryu[1,2,3], Hanna Cho[2], Chiman Song[3], Nam Doo Kim[4], Namkyoung Kim[1,2,3], Taebo Sim[1,2,3,*]

[1]KU-KIST Graduate School of Converging Science and Technology, Korea University, 145 Anam-ro, Seongbuk-gu, Seoul, 02841, Republic of Korea

[2]Severance Biomedical Science Institute, Graduate School of Medical Science, Brain Korea 21 Project, Yonsei University College of Medicine, 50 Yonsei-ro, Seodaemun-gu, Seoul, 03722, Republic of Korea

[3]Chemical Kinomics Research Center, Korea Institute of Science and Technology, 5 Hwarangro 14-gil, Seongbuk-gu, Seoul, 02792, Republic of Korea

[4]Voronoibio Inc., 32 Songdogwahak-ro, Yeonsu-gu, Incheon, 21984, Republic of Korea

[†]These authors contributed equally to this work.

[*]Author to whom correspondence should be addressed.

Table of contents

Table S1. *In vitro* selectivity profiling of **6r** at 1.0 μM against 371 human kinases……………………………………..S2

Table S1. *In vitro* selectivity profiling of **6r** at 1.0 μM against 371 human kinases

kinase	% inhibition	kinase	% inhibition	kinase	% inhibition
RAF1	99.9	CDK19/cyclin C	12.8	PKG1b	3.3
DDR1	99.9	PKCb2	12.7	SYK	3.2
RET	99.4	MAPKAPK2	12.5	CAMK4	3.2
FMS	99.0	MST3/STK24	12.5	CDK7/cyclin H	3.2
c-KIT	98.7	CDK3/cyclin E2	12.4	PKCeta	3.1
FRK/PTK5	98.4	CDK2/cyclin O	12.3	c-MET	2.7
ARAF	98.3	Haspin	12.1	ALK4/ACVR1B	2.6
LYN	97.5	ERK1	11.7	NEK1	2.6
BRAF	97.4	SGK1	11.6	MRCKb/CDC42BP B	2.4
LCK	97.2	MEKK2	11.1	ALK2/ACVR1	2.4
PDGFRa	96.7	MLCK/MYLK	11.0	LATS2	2.1
P38a/MAPK14	96.5	MUSK	10.8	MARK4	2.0
PDGFRb	94.2	MAPKAPK3	10.7	IKKe/IKBKE	2.0
FYN	89.0	TRKB	10.6	JAK2	2.0
FGR	87.7	PKCb1	10.5	IR	1.9
ABL2/ARG	87.2	PLK4/SAK	10.4	RSK4	1.8
LIMK1	87.2	DAPK1	10.1	VRK1	1.6
BLK	84.8	PKG1a	10.1	TEC	1.6
HCK	83.1	PKCd	10.1	LATS1	1.5
RIPK3	82.5	PKMYT1	10.0	DCAMKL1	1.5
EPHA5	78.5	TTBK1	9.9	MASTL	1.3
KDR/VEGFR2	77.8	CDK4/cyclin D3	9.8	NIM1	1.3
FLT4/VEGFR3	77.8	TRKA	9.7	SIK1	1.1
P38b/MAPK11	77.7	STK33	9.5	CK2a2	1.0
EPHA2	77.4	STK25/YSK1	9.3	TYK1/LTK	1.0
YES/YES1	77.0	CHK1	9.3	NEK6	1.0
JAK1	74.9	SRMS	9.2	CDC7/DBF4	0.8
ERBB4/HER4	73.9	COT1/MAP3K8	9.2	CDK16/cyclin Y (PCTAIRE)	0.8
EPHA8	72.3	CDK6/cyclin D1	9.1	PIM2	0.8
c-Src	68.7	PKG2/PRKG2	9.1	SGK3/SGKL	0.8
EPHB1	65.7	SRPK2	9.0	PKCg	0.7
EPHA4	64.7	WNK2	8.7	CDK5/p35	0.7
FLT1/VEGFR1	63.7	CDK5/P25	8.7	STK32C/YANK3	0.6
TAOK2/TAO1	61.9	HIPK4	8.7	DYRK2	0.6

ABL1	60.8	ZAP70	8.7	ROCK2	0.6
LCK2/ICK	60.8	PAK4	8.6	HIPK2	0.5
CK1g1	50.5	PKCiota	8.6	DAPK2	0.3
ZAK/MLTK	50.0	PKN3/PRK3	8.6	HIPK3	0.2
PYK2	48.1	CK1a1L	8.5	ROCK1	0.2
SRPK1	46.3	p70S6K/RPS6KB1	8.4	AKT1	0.0
ALK3/BMPR1A	44.1	PAK3	8.3	ALK	0.0
FGFR2	43.0	CDK2/cyclin E2	8.3	ALK2/ACVR1	0.0
EPHB3	42.5	EPHA1	8.2	ALK3/BMPR1A	0.0
CSK	41.3	CTK/MATK	8.1	ALK4/ACVR1B	0.0
EPHA3	41.1	ERN1/IRE1	8.0	ALK5/TGFBR1	0.0
EPHA7	40.7	CAMK1a	8.0	ASK1/MAP3K5	0.0
EPHA6	39.9	DMPK2	8.0	Aurora A	0.0
ACK1	39.9	MEKK6	8.0	AURORA B	0.0
LIMK2	39.9	TYK2	7.8	BRSK1	0.0
FES/FPS	39.7	JAK3	7.8	CAMK2b	0.0
CK1g2	38.9	FAK/PTK2	7.8	CAMK2d	0.0
EPHB2	38.9	CLK4	7.7	CAMK2g	0.0
CLK2	38.7	CDK2/Cyclin A1	7.6	CDK1/cyclin B	0.0
SLK/STK2	37.8	PAK6	7.6	CDK1/cyclin E	0.0
BRK	37.5	GRK7	7.5	CDK14/cyclin Y (PFTK1)	0.0
TNK1	36.2	GSK3a	7.4	CDK2/CYCLIN A	0.0
p70S6Kb/RPS6KB2	34.0	NLK	7.3	CDK2/CYCLIN E	0.0
RSK2	32.4	MKK7	7.3	CDK8/cyclin C	0.0
CK1G3	32.0	CAMKK1	7.2	CDK9/CYCLIN K	0.0
JNK2	31.8	ULK3	7.2	CDK9/cyclin T1	0.0
BMX/ETK	31.5	BRSK2	7.2	CDK9/cyclin T2	0.0
MSSK1/STK23	30.6	PDK1/PDPK1	7.2	CLK1	0.0
FGFR4	30.1	MEK2	7.1	CLK3	0.0
PKAcg	29.2	WNK3	7.1	DCAMKL2	0.0
LCK	29.0	PKCtheta	6.9	DLK/MAP3K12	0.0
PKD2/PRKD2	28.6	WNK1	6.9	DMPK	0.0
CK1d	27.3	PAK1	6.9	DRAK1/STK17A	0.0
EPHB4	27.3	ZIPK/DAPK3	6.7	DYRK3	0.0
DYRK1B	26.2	SBK1	6.7	DYRK4	0.0
NEK4	25.6	MKK6	6.6	FER	0.0

TIE2/TEK	24.4	ROS/ROS1	6.6	FGFR3	0.0
CK1epsilon	24.2	MEK5	6.5	FLT3	0.0
PKCnu/PRKD3	23.8	STK38L/NDR2	6.5	GRK2	0.0
MLK3/MAP3K11	23.7	ERK5/MAPK7	6.5	GRK3	0.0
RSK1	23.4	MLK4	6.5	GRK4	0.0
TTBK2	23.2	TAOK1	6.1	GRK6	0.0
TNIK	22.4	c-MER	6.1	HGK/MAP4K4	0.0
GCK/MAP4K2	21.8	MKK4	6.0	HPK1/MAP4K1	0.0
CK2a	21.7	GRK5	5.8	IGF1R	0.0
EGFR	21.4	SIK2	5.6	IKKb/IKBKB	0.0
CAMK1g	20.8	STK21/CIT	5.6	IRAK1	0.0
FGFR1	20.7	OSR1/OXSR1	5.5	IRAK4	0.0
BMPR2	20.6	KSR1	5.5	IRR/INSRR	0.0
RIPK2	20.6	RSK3	5.4	KHS/MAP4K5	0.0
CAMKK2	20.5	NEK7	5.4	KSR2	0.0
VRK2	20.1	PAK2	5.4	LKB1	0.0
PHKg1	19.6	CAMK1b	5.4	MAPKAPK5/PRAK	0.0
STK39/STLK3	19.2	ERBB2/HER2	5.4	MARK1	0.0
NEK11	18.3	ALK1/ACVRL1	5.3	MARK2/PAR-1Ba	0.0
TAOK3/JIK	17.8	PKA	5.3	MARK3	0.0
DYRK1/DYRK1A	17.4	TESK1	5.3	MAST3	0.0
STK38/NDR1	17.1	MEK3	5.2	MEKK1	0.0
MELK	17.1	HIPK1	5.2	MINK/MINK1	0.0
MSK2/RPS6KA4	17.1	JNK3	5.1	MLK1/MAP3K9	0.0
TXK	16.8	LRRK2	5.1	MNK1	0.0
GRK1	16.7	CDK18/cyclin Y (PCTK3)	5.1	MNK2	0.0
CAMK2a	16.0	PAK5	5.0	MRCKa/CDC42BPA	0.0
PKCmu/PRKD1	15.9	TBK1	5.0	MSK1/RPS6KA5	0.0
AXL	15.8	PHKg2	5.0	MST2/STK3	0.0
MST4	15.7	MEKK3	4.9	MYLK3	0.0
PKN1/PRK1	15.1	AURORA C	4.9	MYO3b	0.0
TESK2	15.1	PLK3	4.8	NEK2	0.0
ALK6/BMPR1B	15.0	CHK2	4.8	NEK3	0.0
GLK/MAP4K3	15.0	RIPK4	4.8	NEK5	0.0
MAK	15.0	IKKa/CHUK	4.8	NEK9	0.0
MYO3A	15.0	MLCK2/MYLK2	4.8	P38d/MAPK13	0.0

STK22D/TSSK1	14.9	CDK4/cyclin D1	4.7	P38g	0.0
ARK5/NUAK1	14.7	BTK	4.7	PKAcb	0.0
TYRO3/SKY	14.6	MYLK4	4.6	PKCepsilon	0.0
MEK1	14.4	PASK	4.6	PKCzeta	0.0
CAMK1d	14.3	RON/MST1R	4.5	PKN2/PRK2	0.0
CDK3/cyclin E	14.3	RIPK5	4.4	PLK1	0.0
ALK1/ACVRL1	14.2	MST1/STK4	4.3	SIK3	0.0
AKT2	14.0	PBK/TOPK	4.3	SNARK/NUAK2	0.0
LOK/STK10	14.0	CDK6/cyclin D3	4.2	SNRK	0.0
JNK1	14.0	PKCa	4.2	SSTK/TSSK6	0.0
CK1a1	13.7	ERK7/MAPK15	4.2	STK32B/YANK2	0.0
AKT3	13.6	PLK2	3.9	TGFBR2	0.0
PIM1	13.6	CDK17/cyclin Y (PCTK2)	3.9	TLK1	0.0
TRKC	13.6	SGK2	3.8	TLK2	0.0
ERK2/MAPK1	13.5	ITK	3.8	TSSK2	0.0
MLK2/MAP3K10	13.4	CDK1/cyclin A	3.8	TSSK3/STK22C	0.0
ERN2/IRE2	12.9	ULK1	3.7	ULK2	0.0
GSK3b	12.9	STK16	3.6	WEE1	0.0
PRKX	12.8	PIM3	3.4	YSK4/MAP3K19	0.0
TAK1	12.8	MST1/STK4	8.2		
