# Supplementary figures and images for: Identification of Thiazolo[5,4-b]pyridine Derivatives as c-KIT Inhibitors for Overcoming Imatinib Resistance
Source: Cancers (Basel). 2022 Dec 26;15(1):143. doi: 10.3390/cancers15010143 (PMC9817970; doi:10.3390/cancers15010143)

Figure S1

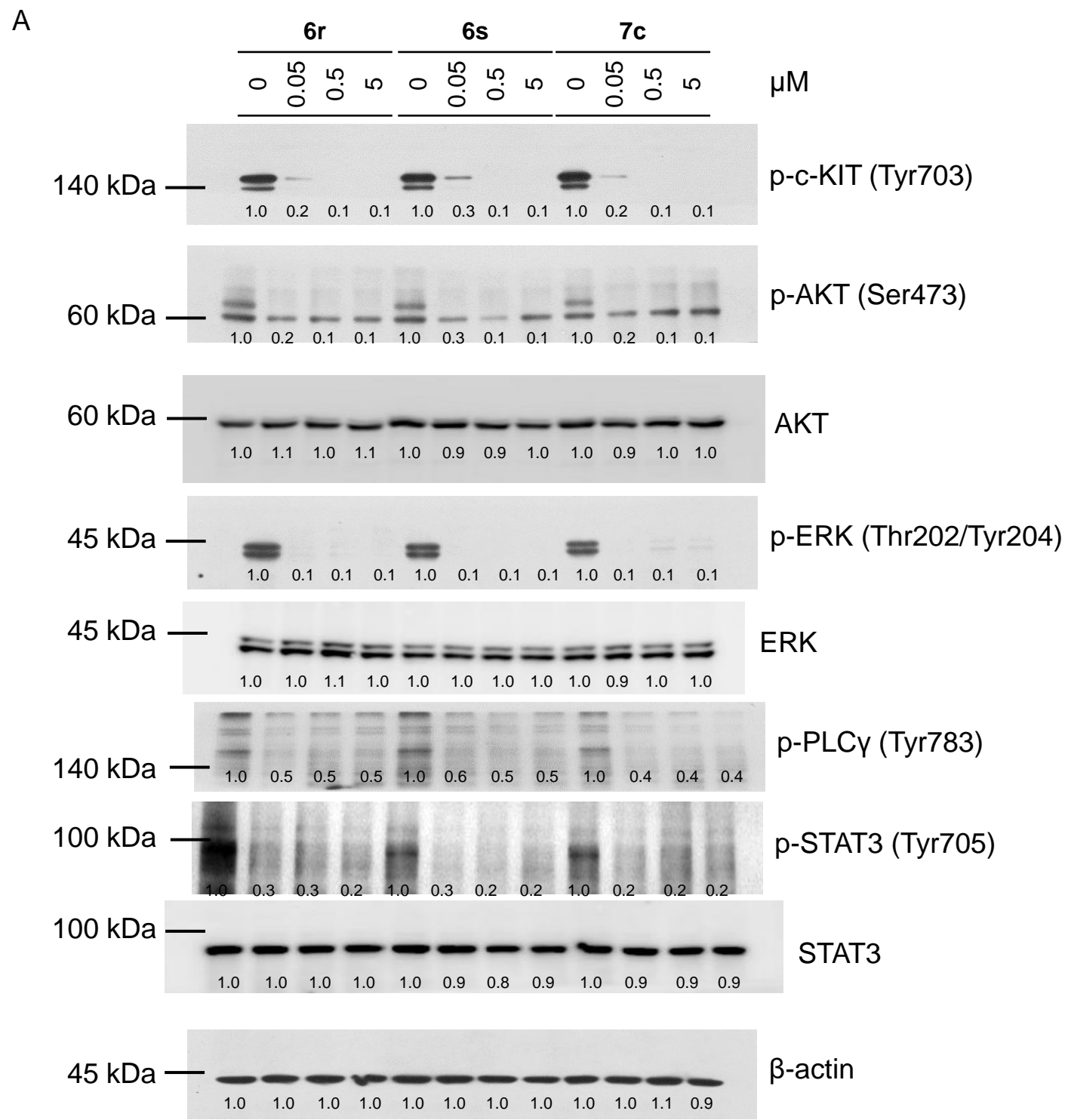

Figure S1

A

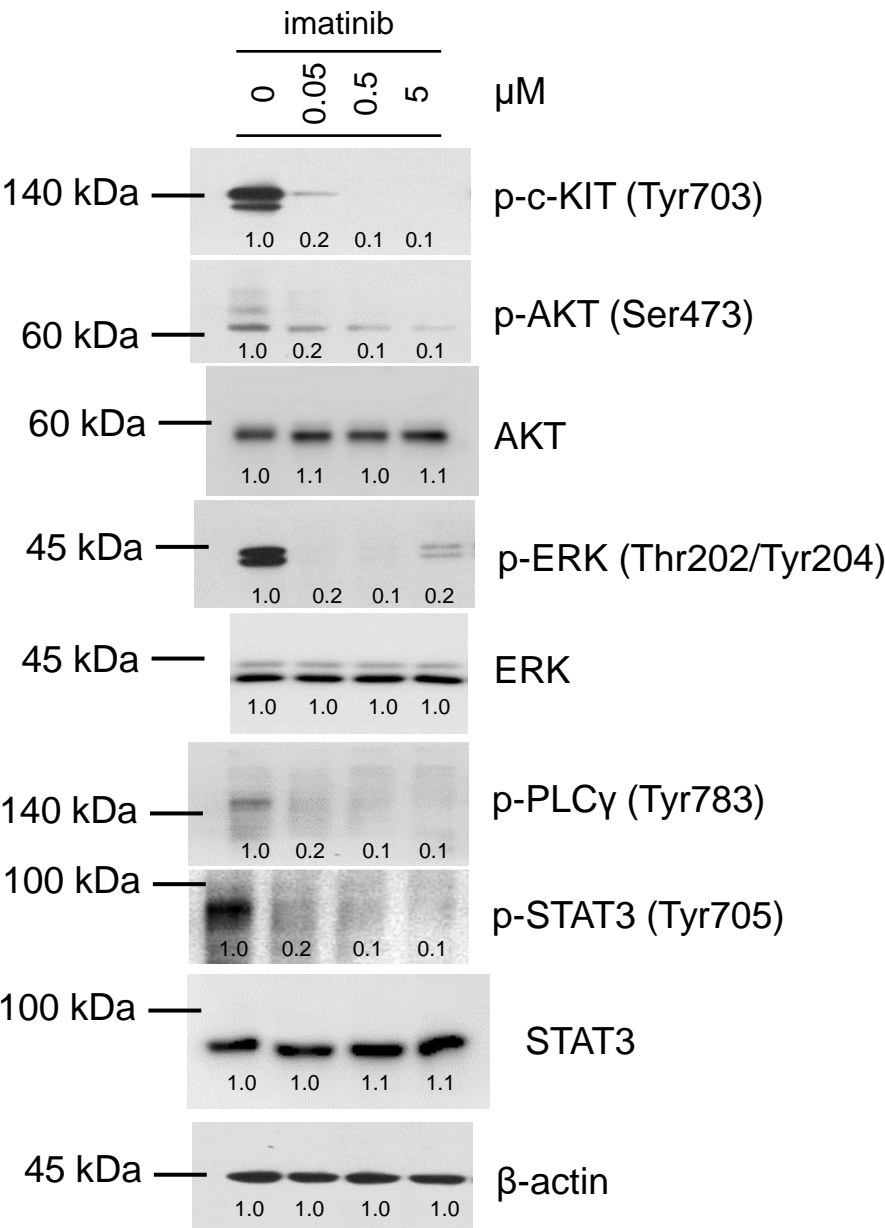

Figure S1

B

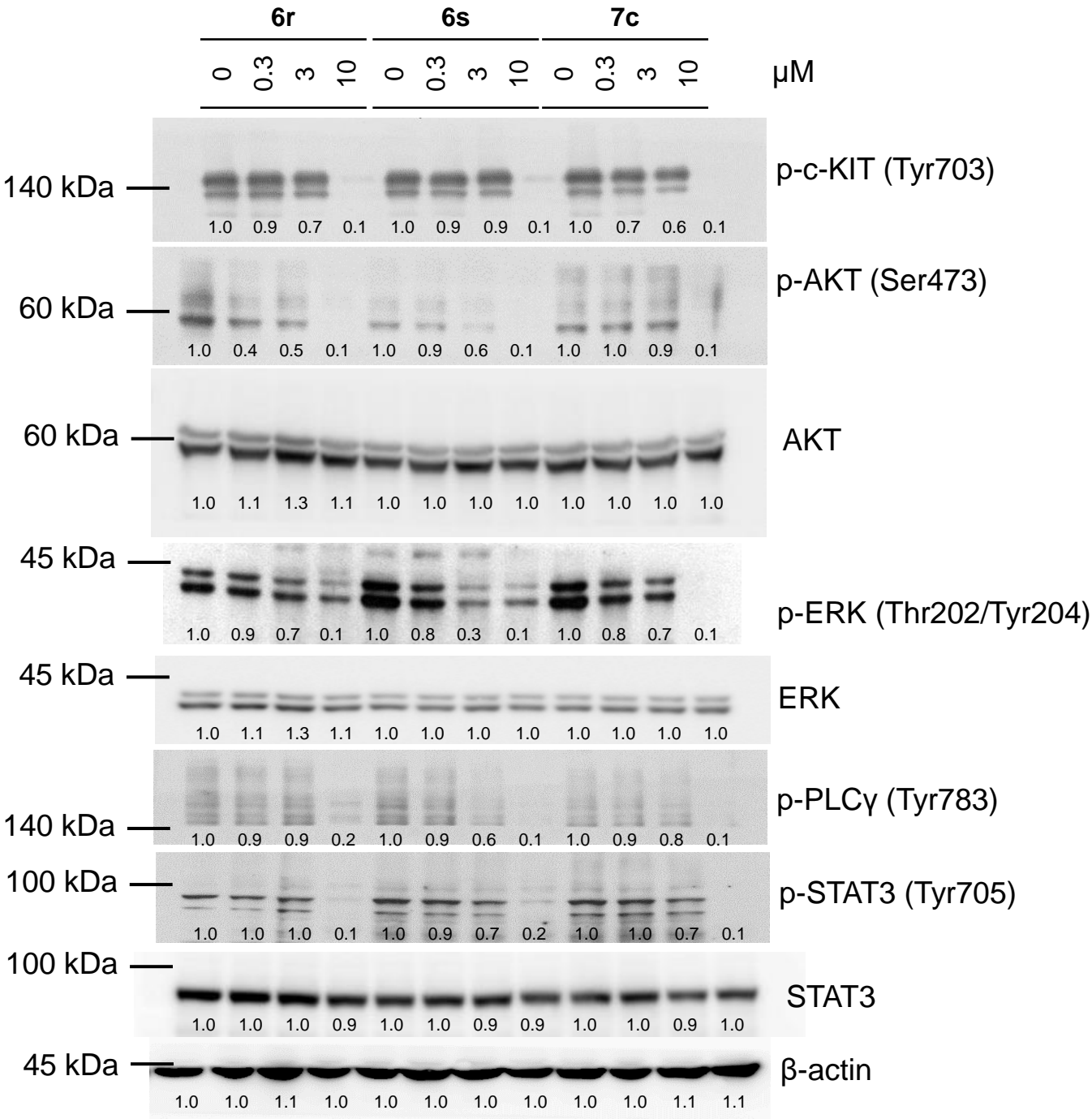

Figure S1

B

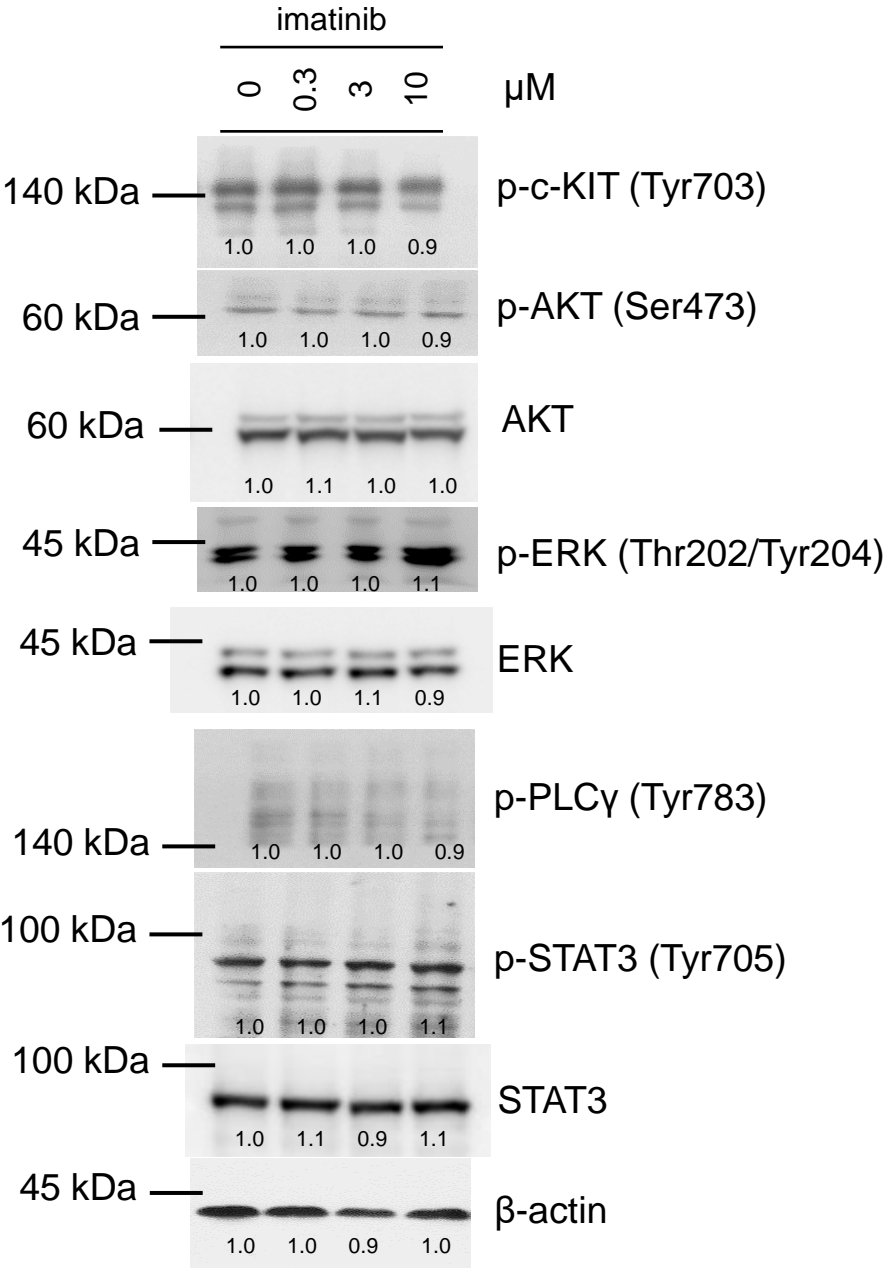

Figure S1

C

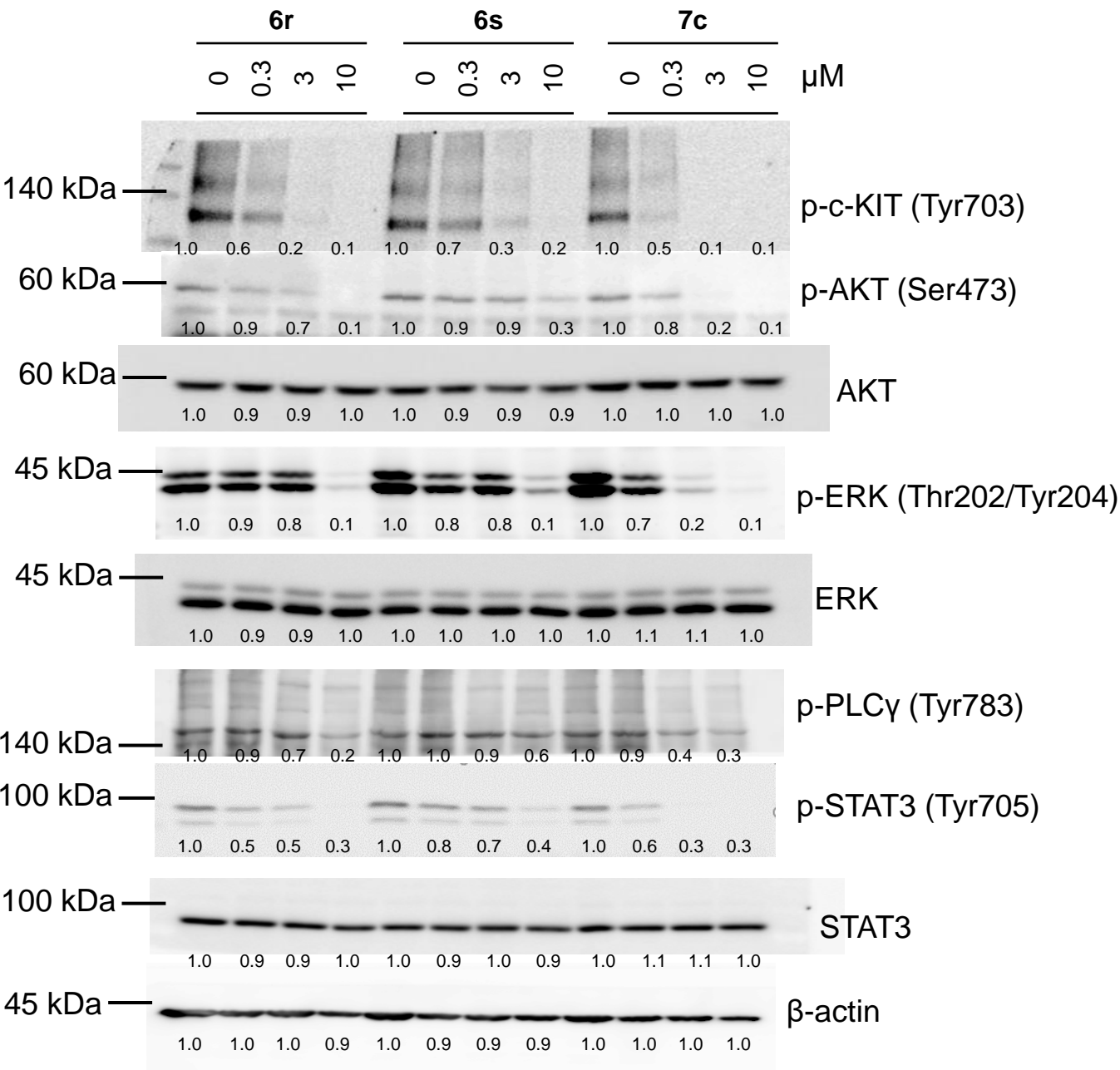

Figure S1

C

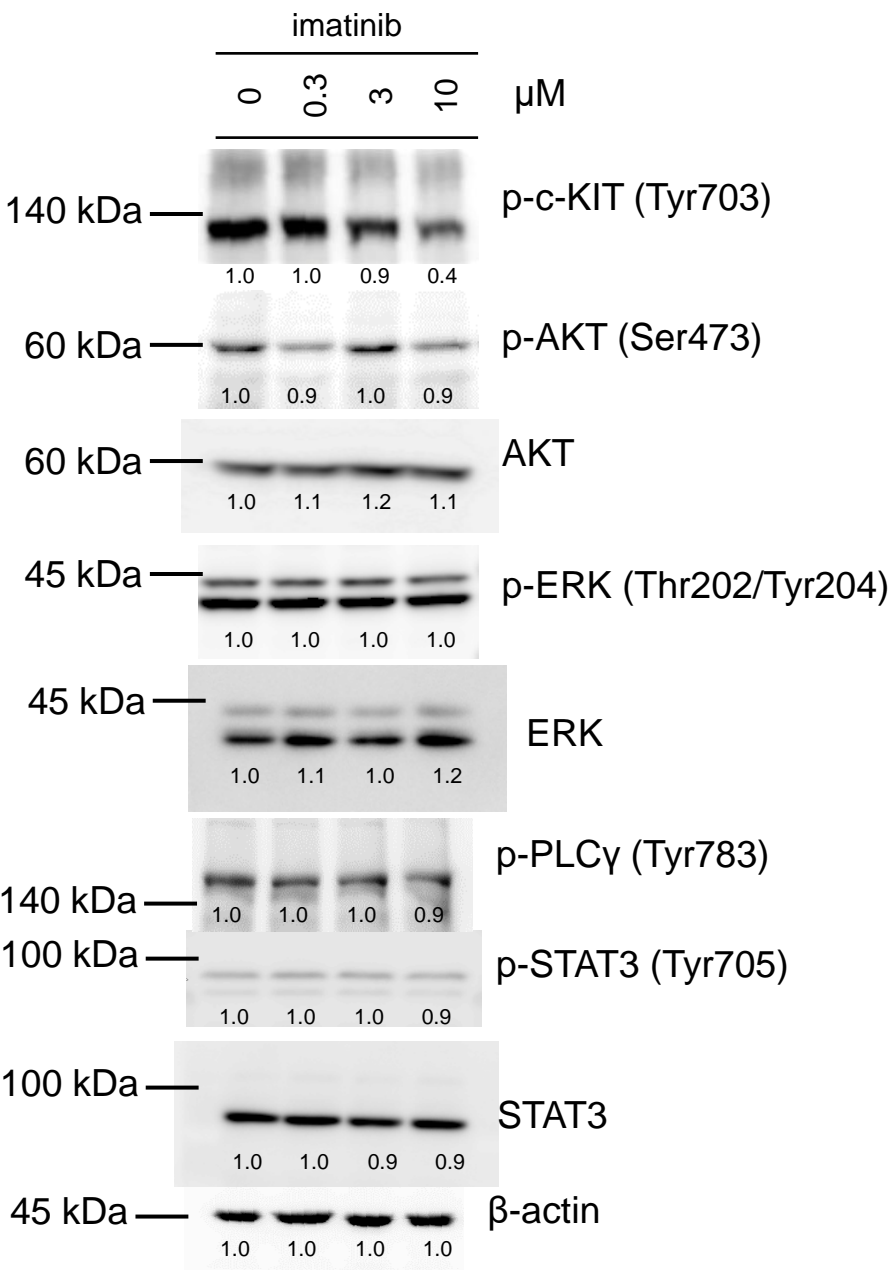

Figure S1

D

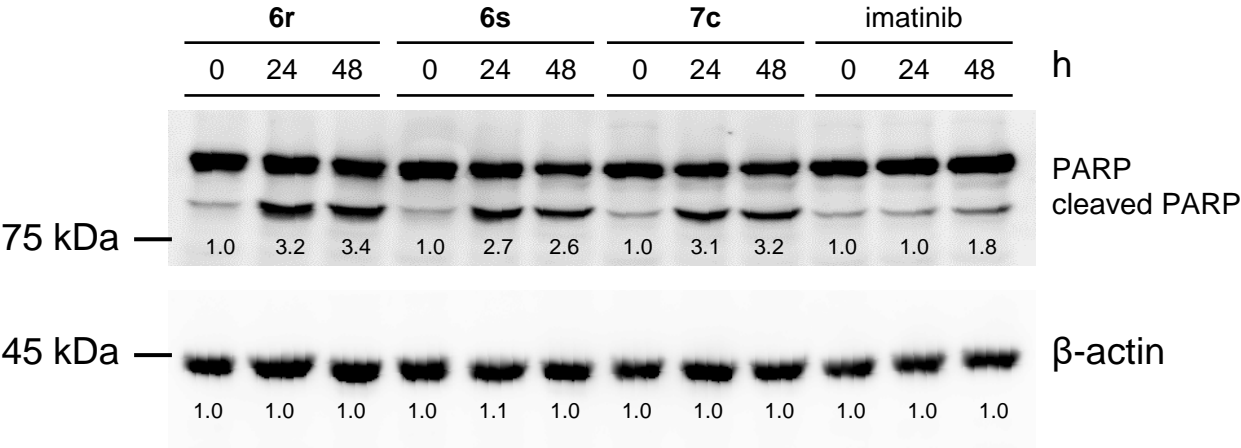

Supplement: Supplementary file 1 [file cancers-15-00143-s001.zip › Figure S1_c-KIT_tbsim_12-26-2022.pdf]
